# Disruption of the tumor suppressor-like activity of aryl hydrocarbon receptor by arsenic in epithelial cells and human lung cancer

**DOI:** 10.7150/ijbs.81423

**Published:** 2023-04-01

**Authors:** Yao Fu, Zhuoyue Bi, Haoyan Ji, Millie Elangbam, Qian Zhang, Yiran Qiu, Wenxuan Zhang, Chitra Thakur, Fei Chen

**Affiliations:** 1 Stony Brook Cancer Center, Department of Pathology, Renaissance School of Medicine, Stony Brook University, Lauterbur Drive, Stony Brook, NY 11794, USA; 2 Department of Pharmaceutical Sciences, Eugene Applebaum College of Pharmacy and Health Sciences, Wayne State University, 259 Mack Avenue, Detroit, MI 48201, USA

**Keywords:** Arsenic, AHR, tumor suppressor, TGFβ, lung cancer

## Abstract

As the most classic and extensively studied transcription factor in response to environmental toxic chemicals, the human aryl hydrocarbon receptor (AHR) has been implicated in mediating some oncogenic responses also. Limited information is available, however, on whether arsenic, a widely presented environmental carcinogen, can regulate AHR to exert its carcinogenic activity. Through chromatin immunoprecipitation and sequencing (ChIP-seq), CRISPR-Cas9 gene editing, RNA-seq, and immunohistochemistry (IHC), in this report we provided evidence showing that arsenic enforces TGFβ and other oncogenic signaling pathways in bronchial epithelial cells through disrupting the tumor suppressor-like activity of AHR. AHR is normally enriched on a number of oncogenic genes in addition to the known phase I/II enzymes, such as genes in TGFβ and Nrf2 signaling pathways and several known oncogenes. Arsenic treatment substantially reduced the binding of AHR on these genes followed by an increased expression of these genes. CRISPR-Cas9-based knockout of AHR followed by RNA-seq further demonstrated increased expression of the TGFβ signaling and some oncogenic signaling pathway genes in the AHR knockout cells. IHC studies on human tissue samples revealed that normal human lung tissues expressed high level of AHR. In contrast, the AHR expression was diminished in the lung cancer tissues. Accordingly, the data from this study suggest that AHR has tumor suppressor-like activity for human lung cancer, and one of the carcinogenic mechanisms of arsenic is likely mediated by the inhibition of arsenic on the tumor suppressor-like activity of AHR.

## Introduction

Environmental arsenic exposure, especially the chronic exposure to inorganic trivalent arsenic (As^3+^) through drinking water or food, is still a major public health concern worldwide. The most common source of environmental As^3+^ is found in groundwater due to the natural deposition of As^3+^ in rocks and sediments 1. Both microbial and chemical reductive dissolutions can release As^3+^ from the arsenic-bearing ferric minerals in the aquifer sediments. In addition, As^3+^ can also be desorbed from iron and aluminum hydroxides under oxidizing, high-pH or other geochemical conditions. A number of hotspots of As^3+^ contamination, the levels of As^3+^ exceed the WHO and US EPA 10ppb guideline, in groundwater were recently documented in western United States, central Mexico, Argentina, the Pannonian Basin, Inner Mongolia, the Indus Valley, the Ganges-Brahmaputra delta, and the Mekong River and Red River deltas 1. It is estimated that about 200-300 million people, including 13 million Americans, are at risk of environmental arsenic exposure 2, 3. The most recent studies in a large population-based prospective cohort with up to 20 years of follow-up has clearly indicated associations of both As^3+^ exposure and As^3+^ metabolism with the increased risk of cancer and other disease mortalities 4. Meanwhile, geospatial mapping revealed a strong correlation between higher blood As^3+^ concentration and elevated cancer incidences in some specific geographic regions 2. Although cautions should be taken for some inconsistencies, ecological analysis unraveled connections between low-moderate levels of drinking water arsenic concentrations and lung or bladder cancer incidence in U.S. counties 5. This notion was independently supported by the findings in a retrospective study in Chile that showed latency effects of As^3+^ exposure and the increased mortality of lung cancer, bladder cancer and kidney cancers 40 years after exposure reduction 6. Furthermore, a more comprehensive U.S.-based prospective investigation in the cohort of Strong Heart Study (SHS) provided unequivocal evidence suggesting contribution of low-moderate environmental As^3+^ exposure to the increased mortality for cancers of the lung, prostate, and pancreas, but not the cancers of esophagus, stomach, colon, rectum, and breast 7. Histologically, As^3+^ might be the first identified human carcinogen by Paracelsus in 16th century who described that inhaled arsenic-containing ore dust caused “Mala Metallorum”, the “Schneeberger Lung Disease” or lung cancer among the miners or metal refiners 8. The carcinogenic property of As^3+^ had been confirmed and/or validated in a wide range of experimental models. As^3+^ is highly capable of inducing oxidative stress, protein kinase and transcription factor activation, metabolic and epigenetic reprogramming, genotoxicity, and the generation of the cancer stem-like cells 9, 10.

The aryl hydrocarbon receptor (AHR), a basic helix-loop-helix-PER-ARNT-SIM transcription factor and environmental sensor, had been extensively studied in the toxicity, immunity and carcinogenicity of environmental toxicants and carcinogens 11. As one of the subclasses of nuclear receptors that is conserved in almost all animal species 12, AHR is traditionally considered as the most important regulator of drug and xenobiotic metabolism through direct interactions with a broad spectrum of lipophilic small molecules, including dioxins, polycyclic aromatic hydrocarbons, polyphenols, and tryptophan derivatives found in environment, diet, bacterial metabolites, and the processes of physical metabolism. Cross-talk or synergy of AHR with Nrf2, HIF1α and other stress-response signaling pathways had been well-documented in the AHR-mediated biological responses. In our recent ChIP-seq analysis of the As^3+^-treated bronchial epithelial cell line BEAS-2B cells, we noted As^3+^-induced Nrf2 binding peaks in the AHR gene, suggesting Nrf2 dependency of the AHR expression in cellular response to As^3+^ 10. Since its ubiquitous expression in immune cells, esp. in T helper 17 (Th17) cells and T regulatory (Treg) cells, the significant role of AHR in adaptive immunity had been recognized for more than two decades 13. AHR is able to enforce FoxP3 expression for the functional specialization of the Treg cells 14. AHR is also capable of facilitating the lineage development of the Th17 cells by antagonizing the negative regulators for Th17 cell differentiation 15. These effects of AHR on T cells may also contribute to the host-microbiota interactions. Emerging evidence suggests that microbiome produces multiple endogenous ligands or agonists to alter the activity of AHR and other nuclear receptors that linked to the maintenance of mucosal Treg and Th17 cells 16, and vice versa, both Treg and Th17 cells may influence the composition and function of the intestinal microbiota 17.

Since the first identified AHR agonist, 2,3,7,8‐tetrachlorodibenzo‐*p*‐dioxin (TCDD), is a known human carcinogen and some reports indicated carcinogenic-like roles of AHR in several experimental models, AHR has long been viewed as an oncogenic transcription factor. However, under many different circumstances, activation of AHR may be tumor suppressive 11. In fact, TCDD activated AHR has been shown to be inhibitory for stemness transcription factors SOX2, Nanog and SALL4, which are also important in the self-renewal of the cancer stem-like cells, during the osteogenic process of the mesenchymal stem cells (MSCs) 18. Several earlier studies tested the activation of AHR by the known carcinogen, As^3+^, with a very diversified conclusions, possibly due to the use of different cell lines or experimental models 19-21. In the present study, we investigated whether AHR is one of the key mediators for the carcinogenic effect of As^3+^ in human bronchial epithelial cells. Unexpectedly, our findings imply that AHR itself possesses tumor suppressor-like property under the basal condition, and the carcinogenicity of As^3+^ is partially achieved through antagonizing the transcriptional activity of AHR. These data, thus, may provide new insights into the prevention and treatment of human malignancies associated with the exposure to environmental As^3+^.

## Materials and Methods

**Cells and Reagents** — The human bronchial epithelial cell line BEAS-2B was purchased from ATCC (Manassas, VA). BEAS-2B cells were cultured in Dulbecco's Modified Eagle's Medium-high glucose medium (DMEM) (Sigma-Aldrich, St. Louis, MO) supplemented with 5% (v/v) fetal bovine serum (FBS) (R&D system, Minneapolis, MN), 1% (v/v) penicillin-streptomycin and 1% (v/v) L-Glutamine (Thermo Fisher Scientific, Waltham, MA) at 37℃ humidified incubator with 5% CO_2_. Arsenic was purchased from Sigma in the format of AsCl_3_ (Sigma-Aldrich, St. Louis, MO).

**Western Blotting** — The total cell lysates were prepared in 1x RIPA buffer from Cell Signaling (Danvers, MA) supplemented with protease and phosphatase inhibitor cocktail and 1mM PMSF (Thermo Fisher Scientific, Waltham, MA). Cell lysates were collected and sonicated in 4℃ with 1.0 sec on and 0.7 sec off pulses at 12% power amplitude followed by incubation in 4℃ for 30 min. Cell lysates were centrifuged at 12,500 rpm for 15 min and supernatant was collected for the protein concentration determination with BCA Protein Assay Reagent kit (Thermo Fisher Scientific, Waltham, MA). Protein samples were prepared using 4x LDS Sample Buffer and dithiothreitol with the final concentration of 50 mM and denatured at 98℃ for 10 min. Protein samples were separated into SDS-PAGE gels and then immediately transferred to the PVDF membrane (MilliporeSigma, Burlington, MA). Transferred membrane was blocked with 5% non-fat milk in TBST at room temperature for 1 hour and incubated with diluted primary antibody at 4℃ overnight. Membrane was washed 3 times for 10 min each next day and incubated with diluted secondary antibody. Membrane was then incubated in ECL Western Blotting Substrate (Thermo Fisher Scientific, Waltham, MA) for 5 min at room temperature and visualized in ChemiDoc Imaging System (Bio-Rad, Hercules, CA).

**Nuclear Protein Extraction** — One and a half million BEAS-2B cells were seeded in T75 flask overnight and treated with 0 or 4 µM As^3+^ for 6 h. Nuclear and cytoplasmic protein was extracted using Nuclear Extraction Kit (Abcam, Cambridge, United Kingdom) according to the protocol provided by the manufacturer. Gene expression was tested by Western blotting as described previously. Anti-H3 antibody (Abcam, Cambridge, United Kingdom) and anti-tubulin antibody (Cell Signaling, Danvers, MA) were used as internal calibration for protein loading of nuclear proteins and cytoplasmic proteins, respectively.

**AHR Luciferase Reporter Assay** — AHR Luciferase Reporter Assay was performed with The Human Aryl Hydrocarbon Receptor (AHR) assay kit (Indigo Biosciences, State College, PA) according to the protocol from the manufacturer. Luciferase reporter cells were the gene engineered Huh7 cells provided by the manufacturer that luciferase reporter gene was inserted in the downstream of dioxin/xenobiotic responsive elements (DRE/XRE) without introduction of other transcription factor binding elements. Briefly, 200 µl of reporter cells was dispensed into each well and pre-incubated for 5 h, followed by the treatment of different concentrations of As^3+^. Bioluminescence was detected by Promega GloMax plate reader.

**Chromatin Immunoprecipitation Sequencing Analysis (ChIP-seq)** — One million cells were seeded in T75 flasks and starved with serum-free medium overnight when the confluency reached to 90%. Following the starvation, cells were treated with 1 µM As^3+^ for 6 h before fixing by fresh specially formulated Formaldehyde Solution. After fixation, DNA was sheared into fragments and then incubated with ChIP-validated AHR antibody (Enzo Life Sciences, Farmingdale, NY). Once the immunoprecipitation with anti-AHR antibody is complete, the DNA that was immunoprecipitated was analyzed. Seventy-five-nucleotide single-end (SE75) sequence reads were generated by NextSeq 500 and mapped to the genome. Then the aligned reads, also known as tags, were extended to 150-250 bp fragments with Active Motif software and the fragment density was determined by the number of fragments in each 32-nucleotide bin. Last MACS/MACS2 and SICER methods were applied to find the tags enriched in the ChIP/IP data when compared to the Input data, and the tag number of all samples were normalized and analyzed. Raw ChIP-seq data of AHR can be accessed in NCBI Gene Expression Omnibus (GEO) with accession number of GSE214840.

**Real-time PCR** — Total RNA was extracted by RNeasy Plus Mini Kit (Qiagen, Germantown, MD) according to the protocol provided by the manufacturer. RNA concentration and purity were measured by nanodrop (Thermo Fisher Scientific, Waltham, MA). One µg RNA was converted to cDNA with High-Capacity cDNA Reverse Transcription Kit (Applied Biosystems), followed by adding 2.5 µl of 10x diluted cDNA to Fast SYBR Green Master Mix reaction system (Applied Biosystems, Waltham, MA) and mRNA expression was measured in Roche LightCycler 480 Instrument.

**CRISPR-Cas9 Plasmid Construction and Transformation** — To design single guide RNA, AHR exon 1 and exon 2 were input into online sgRNA design tool (https://chopchop.cbu.uib.no/ and http://crispor.tefor.net/). The output sgRNA-1 5'-TCACCTACGCCAGTCGCAAGCGG-3' and sgRNA-2 5'-AGCGGCATAGAGACCGACTT-3' were used to construct CRISPR-Cas9 plasmid. sgRNA and its corresponding reverse complementary strand were synthesized and annealed at 95 ℃ for 5 min and cooled down to 25 ℃ at the speed of 5 ℃/min. Vector pSpCas9-2A-Blast (Addgene, Watertown, MA) was linearized and dephosphorylated by restriction enzyme Bpil and phosphatase FastAP (Thermo Fisher Scientific, Waltham, MA) at 37℃ for 30 min. 200x diluted annealed sgRNA and linear plasmid were ligated by T4 ligase (Thermo Fisher Scientific, Waltham, MA) at room temperature for 30 min. The ligation product was then transfected into competent cells DH5α (Thermo Fisher Scientific, Waltham, MA) according to the protocol provided by the manufacturer. Plasmid was extracted with QIAprep Spin Miniprep Kit and sent to Genewize to verify sgRNA was inserted successfully and correctly.

**Transfection and Blasticidin Selection** — BEAS-2B cells were seeded in each well of 6-well plate at the number of 2x 10^5^ and cultured overnight. Five hundred ng CRISPR plasmid [pSpCas9(sgAhR)], 200 µl of Opti-MEM I Reduced Serum Medium (Thermo Fisher Scientific, Waltham, MA) and 1.5 µl of Lipofectamine 2000 (Invitrogen, Waltham, MA) were added into each well according to the protocol provided by the manufacturer. After 48 h, cells were sub-cultured into 10 cm petri dish and cultured for 24 h. Next day cell culture medium was replaced by DMEM medium supplemented with 10 µg/ml Blasticidin for 2 weeks cell selection. Colonies were picked up and screened by Western Blotting to detect AHR expression. Colonies that have no AHR expression were designed as knockout (KO) cells, while have AHR expression were designed as wildtype (WT) cells.

**Immunohistochemistry** — Tissue microarray slides purchased from US Biomax were processed for immunohistochemistry (IHC) staining for AHR protein. Tissue slides were deparaffinized with xylene and hydrated in a series of alcohol gradients, followed by the incubation with 1.5 to 3% H_2_O_2_ in PBS at room temperature for 20 min. Following the incubation, slides were heated in citrate buffer with pH 6.0 (Thermo Fisher Scientific, Waltham, MA) in a microwave for 20 min to retrieve antigen. To prevent non-specific binding of antibodies, slides were incubated in blocking buffer (5% goat serum, 0.2% Triton X-100 in PBS) at room temperature for 2 h. Primary antibody against AHR (Enzo Life Sciences, Farmingdale, NY) was 1:100 diluted in blocking buffer and slides were incubated at 4℃ overnight. Next day slides were incubated in 1:200 diluted goat-anti-rabbit biotinylated secondary antibody at room temperature for 2 h. IHC signal was developed by the incubation with avidin-biotin complex (ABC) reagent (Vector Laboratories, Newark, CA) at room temperature for 45 min and 3,3'-Diaminobenzidine (DAB) reagent. Stained tissue was counterstained with nuclear counterstain hematoxylin (Sigma-Aldrich, St. Louis, MO) and mounted with Entellan (Electron Microscopy Sciences, Hatfield, PA). Images were captured under the bright field of Nikon Eclipse Ti-S Inverted Microscope.

**RNA sequencing (RNA-seq) and Single-cell RNA Sequencing (scRNA-seq)** — Both RNA-seq and scRNA-Seq was used to determine the expression profiles of the WT and AHR KO cells. One million BEAS-2B AHR WT and KO cells were seeded in 10 cm petri dish. When the confluency reached 70-80%, cells were starved with serum-free medium overnight and treated with 1 µM As^3+^ for 6 h. Cells were harvest according to the Sample Preparation Demonstrated Protocol provided by 10x Genomics (Pleasanton, CA). Libraries for single cells were generated using the 10x Chromium System (Chromium Next GEM Single Cell 3' Library Kit v3.1). Libraries were quantified using a Qubit 2.0 fluorimeter and Qubit dsDNA BR Assay kit (Thermo Fisher Scientific, Waltham, MA) and run on an Agilent TapeStation 2200 for quality control. Libraries were run on NovaSeq 6000 (50,000 reads/cell; single index). 10x Genomics Cell Ranger 6.0.1 mkfastq was used to demultiplex reads by sample before the count parameter was used to align reads (refdata-cellranger-GRCh38-3.0.0) and tabulate gene counts per cell.

**Statistics** — SigmaPlot and Student's t-test were used for determining statistical significance of all quantitative data that were expressed as mean ± standard deviation (SD). The statistical significance of the AHR positivity rate in immunohistochemistry of AHR among normal human lung tissues and human lung cancer tissues was determined by Fisher exact probability test. Reads Per Kilobase of transcript per Million (RPKM) was used for normalization and comparison of RNA-seq/scRNA-seq. Comparative analysis and normalization of ChIP-seq were performed as described previously 10. Publicly available databases, TNMplot and Kaplan-Meier Plotter, were used for gene expression and patient survival probability analyses, respectively.

## Results

**Figure 1:**
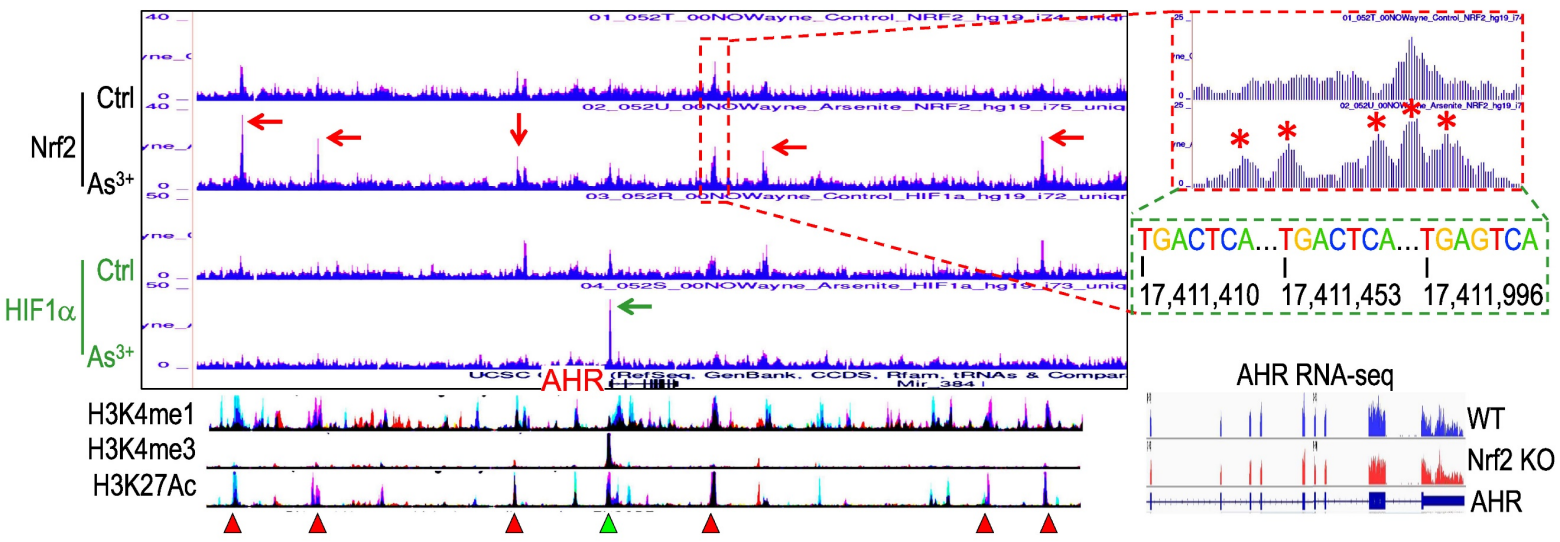
Contribution of Nrf2 to the As^3+^-induced AHR expression. ChIP-seq showed enhanced enrichments of Nrf2 (pointed by red arrows) and HIF1a (pointed by green arrow) in the BEAS-2B cells treated with 1 µM As^3+^ for 6h. Bottom spectrums of H3K4me1, H3K4me3 and H3K27Ac are extrapolated from UCSC Genome Browser for Human GRCh38/hg38. The Nrf2 enrichment peak at the proximal downstream of AHR gene is magnified on the right with conserved Nrf2 binding element sequences, TGACTCA/TGAGTCA. Numbers below the Nrf2 elements indicate the relative genomic positions of these Nrf2 elements in human GRCh37/hg37. Red triangles denote super enhancer regions featured with elevated levels of H3K4me1 and H3K27Ac. Green triangle points to the H3K4me3-enriched promoter of AHR gene. Bottom right panel shows RNA-seq spectrums of the AHR transcripts in the wild-type (WT) BEAS-2B cells and the Nrf2 KO BEAS-2B cells, respectively.

**Nrf2 contributes to As^3+^-induced AHR expression.** We had recently performed ChIP-seq for Nrf2 as well as HIF1α in the human bronchial epithelial cells BEAS-2B with or without 1 μM As^3+^ treatment for 6h, and found many genes related to metabolism and oncogenesis were up-regulated by As^3+^ in a Nrf2 dependent manner 10. Since the coordination between Nrf2 and AHR in xenobiotic responses, we also checked the status of Nrf2 enrichment in the gene locus of AHR. Several Nrf2 enrichment peaks were noted in the upstream and downstream of the AHR gene, which were significantly enhanced in the cells treated with As^3+^ (Figure 1). As^3+^ treatment also induced a pronounced enhancement of HIF1α in the promoter region of the AHR gene. By comparing to the known histone markers of super enhancer, H3K4me1 and H3K27ac, all these Nrf2 enrichment peaks are overlapped with the potential super enhancers in the AHR gene locus. Detailed survey of the consensus Nrf2 binding elements revealed most of these Nrf2 enrichment peaks contain the conserved Nrf2 element TGACTCA/TGAGTCA with one or two nucleotides' differences. The Nrf2 peak in the proximal downstream of the AHR gene contains three consecutive Nrf2 binding elements, which suggested the likelihood of Nrf2 contribution to the As^3+^-induced AHR gene expression. Indeed, RNA-seq showed measurable decrease of AHR transcription in the Nrf2 gene knockout cells (Figure 1, bottom right).

**Figure 2:**
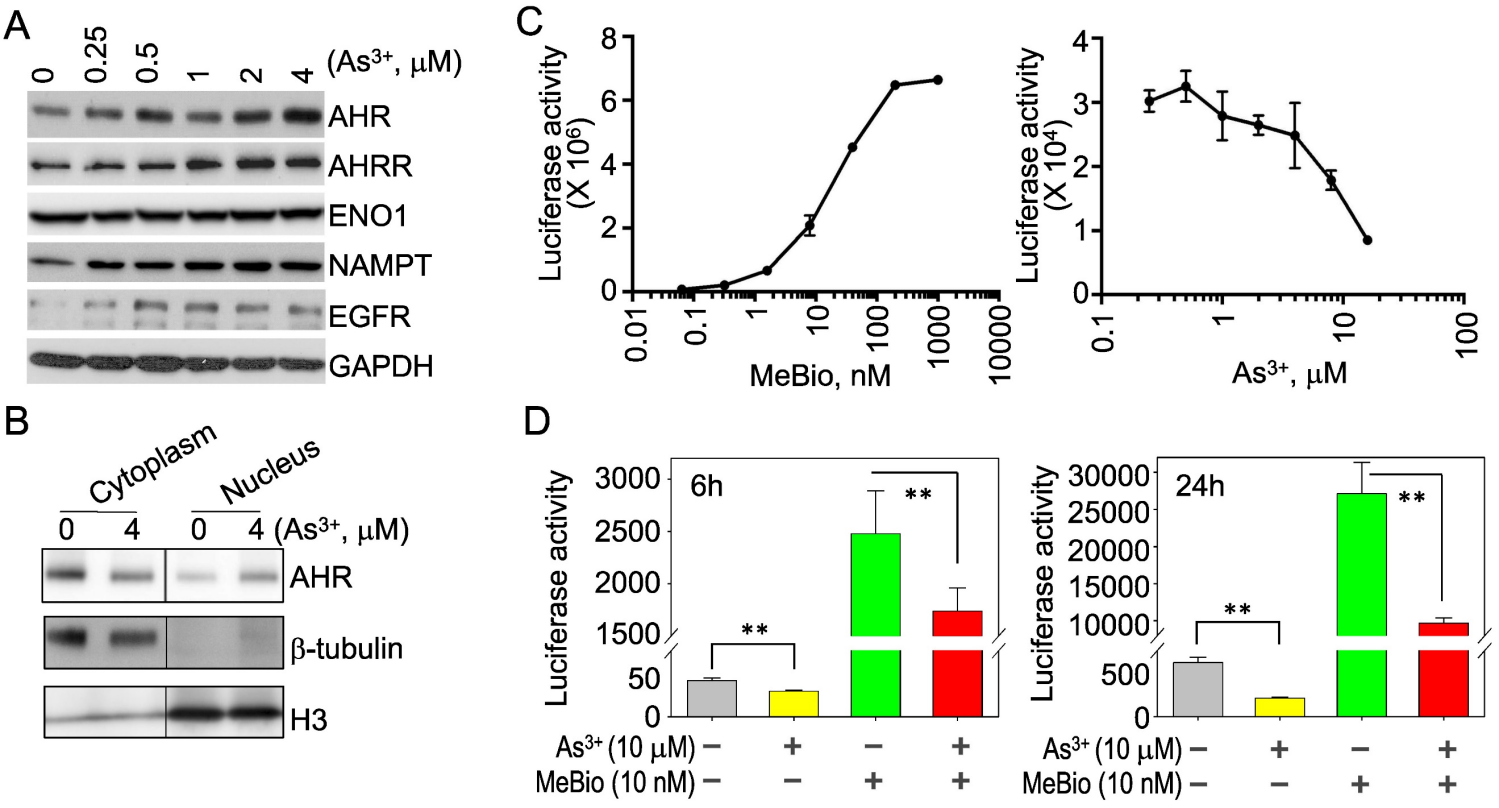
As^3+^ inhibits the transcriptional activity of AHR. (A) BEAS-2B cells treated with the indicated concentrations of As^3+^ for 6h, followed by Western Blotting for AHR, AHRR, ENO1, NAMPT, EGFR, and GAPDH. (B) Fractionation of the cytosolic and nuclear proteins of the BEAS-2B cells with or without As^3+^ treatment for 6h. Protein levels of AHR, b-tubulin and histone H3 were determined by Western Blotting. (C) AHR dependent luciferase reporter cells were treated with MeBio as a positive control or As^3+^ for 6h, followed by AHR luciferase activity assay. (D) As^3+^ represses basal and MeBio-induced AHR luciferase reporter gene activity in the reporter cells treated for 6h (left) or 24h (right). **: p < 0.05.

**Opposite regulation of As^3+^ on the activation and activity of AHR**. To verify that As^3+^ is truly able to induce AHR, we treated the BEAS-2B cells with different concentrations of As^3+^ from 0.25 to 4 μM for 6h and observed a dose-dependent induction of the AHR protein by As^3+^ (Figure 2A). As^3+^ is also able to induce the protein accumulation of AHRR, NAMPT and EGFR (Figure 2A). By fractionation of the cytoplasmic and nuclear proteins of the control and As^3+^-treated cells, we noticed a moderate increase of nuclear translocation of the AHR protein in cellular response to As^3+^ (Figure 2B), which indicated an activation of AHR by As^3+^. To determine whether As^3+^ can upregulate the activity of AHR also, we next performed AHR-dependent luciferase activity assay for the reporter cells treated with MeBio, an analog of 6-bromoindirubin-3'-oxime as a positive control for AHR activity, or As^3+^ for 24h. MeBio is very potent in inducing AHR reporter gene activity, even at a very lower range of concentration (Figure 2C, left panel). There is no induction of AHR reporter gene activity by As^3+^ at every concentration point tested (Figure 2C, right panel). In contrast, a dose-dependent inhibition of As^3+^ on the AHR reporter gene activity was observed at the range of 1 to 16 μM. This inhibition of As^3+^ on AHR transcriptional activity was further confirmed in a co-treatment of the reporter cells with 10 μM As^3+^ and 10 nM MeBio for 6h and 24h, respectively. Again, As^3+^ along inhibited the AHR reporter activity at both time points. Addition of As^3+^, moreover, substantially reduced the AHR transcriptional activity induced by MeBio (Figure 2D). These data, accordingly, suggest that despite the detectable induction of AHR protein, As^3+^ is a potent inhibitor for the transcriptional activity of AHR.

**Figure 3:**
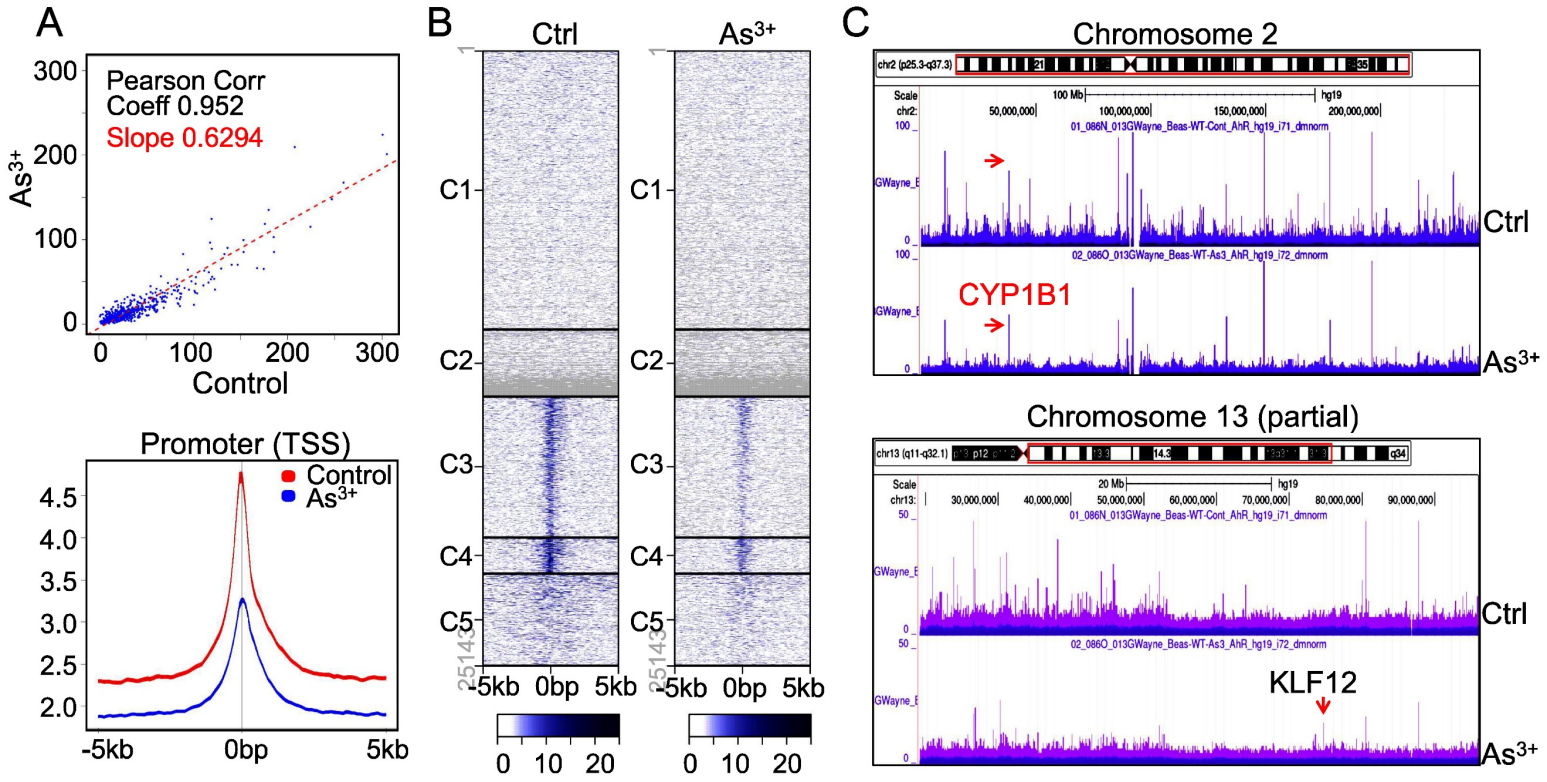
As^3+^ impedes chromatin binding of AHR. (A) AHR peak correlation in ChIP-seq between the control and the cells treated with 1 µM As^3+^ for 6 h (top panel) and Average plots of AHR peaks across the promoter region of the genes on the genome. (B) Heatmaps of AHR enrichment peaks on the promoter region of the defaulted five gene clusters. (C) Genome browser screenshot of the AHR enrichment peaks from ChIP-seq for chromosome 2 and chromosome 13. Except KLF12 (pointed by red arrow), the heights of all AHR peaks are shortened in the cells treated with As^3+^.

**As^3+^ diminishes AHR binding on chromatin.** The downregulation of AHR transcriptional activity by As^3+^ is indicative that As^3+^ reduces the DNA binding of AHR on the genome. To test this hypothesis, we conducted ChIP-seq to profile the global binding of AHR on the genome in both control cells and the cells treated with 1 μM As^3+^ for 6h. A pronounced decrease of AHR occupancy on the genome (Figure 3A, upper panel) as well as the promoters of genes (Figure 3A, bottom panel) was noted in the cells treated with As^3+^, which explains the diminished transcriptional activity of AHR by As^3+^ as indicated in Figure 2. Heatmap of ChIP-seq showed a clear decrease of AHR enrichment in the promoters of gene clusters 3, 4 and 5 (Figure 3B). This is also true in the visualization of the Genome Browser map of the entire chromosome region (Figure 3C, upper panel, exampled by chromosome 2) or partial region of chromosomes (Figure 3C, bottom panel, exampled by the partial region of chromosome 13 long arm). In both examples, the heights of AHR enrichment peaks are significantly lowered in the cells treated with As^3+^ relative to the control cells.

**Figure 4:**
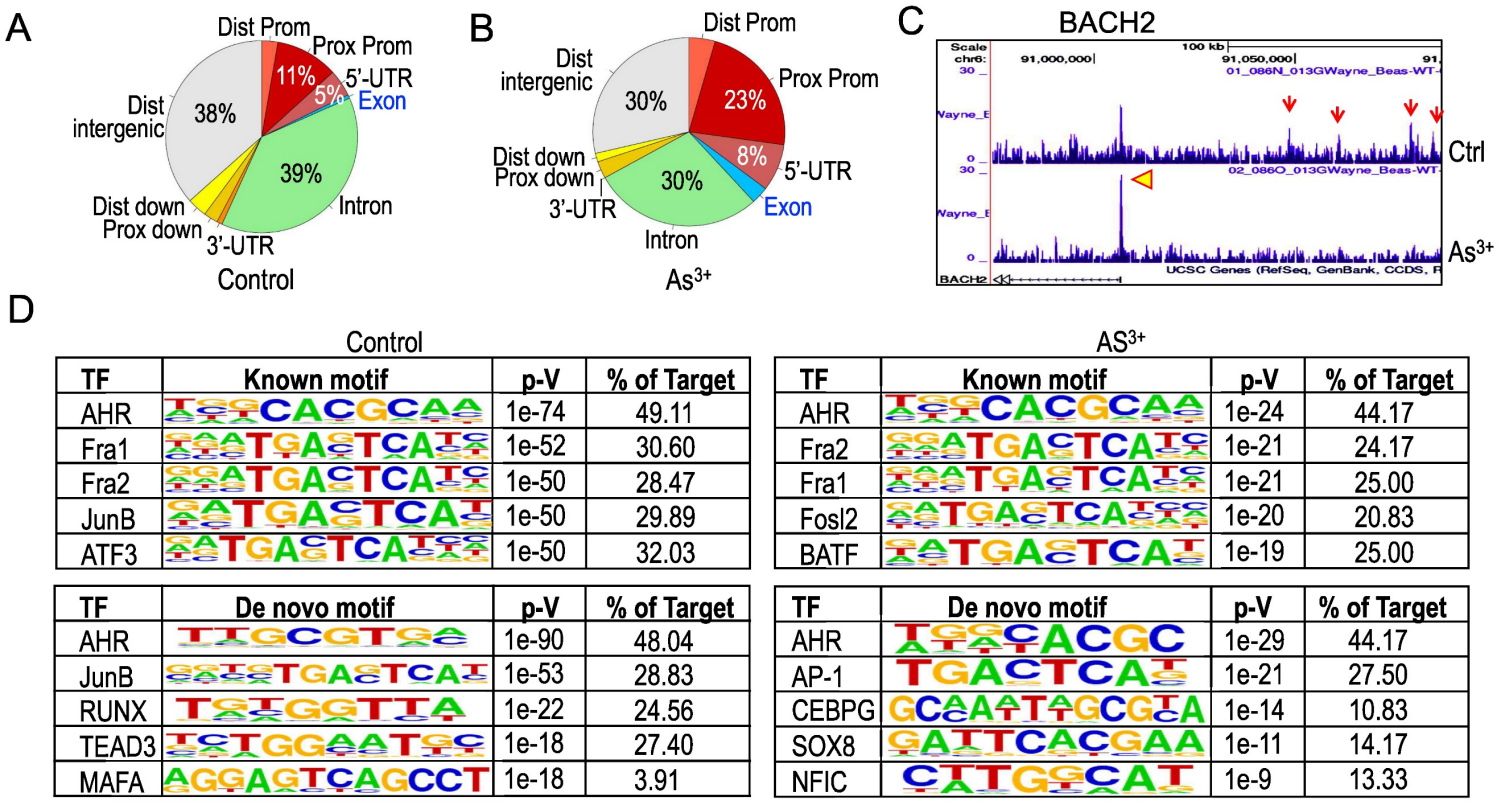
Changed genomic distribution of AHR enrichment in the As^3+^-treated cells. (A & B) Piecharts of AHR enrichment peaks in the indicated genomic regions of the control (A) and As^3+^-treated cells (B). Dist: distal; Prox: proximal; Prom: promoter. (C) Genome browser screenshot showing the pattern of AHR enrichment peaks on the BACH2 gene locus. Red arrows denote AHR peaks in the distal intergenic region; yellow arrowhead indicates the AHR peak in the promoter region of BACH2 gene. (D) HOMER Known and *De Novo* Motif search using the findMotifsGenome program for the 200bp surrounding the apex of AHR peaks in ChIP-seq.

**Treatment of the cells with As^3+^ shifts distribution of AHR binding on the genome.** To get additional insights into how As^3+^ alters AHR binding on the genome, we next analyzed genome wide distribution of the AHR binding peaks in both control cells and the cells treated with 1 μM As^3+^ for 6h. Under the basal condition, the AHR enrichment peaks as determined by ChIP-seq in the intron, distal intergenic, proximal promoter, and 5-UTR regions are accounted for 39%, 38%, 11%, and 5%, respectively (Figure 4A). Treatment of the cells with As^3+^ diminished AHR binding in the intron and distal intergenic region to 30% each, but increased enrichment peaks of AHR in the proximal promoter and 5-UTR regions (Figure 4B). In addition, a marginal increase of AHR peaks was noted in the distal promoter and exon regions in the cells treated with As^3+^. Such a shift of AHR enrichment peaks was exampled by the AHR peaks on the BACH2 gene encoding a BTB domain basic leucine zipper transcription factor important for cell apoptosis and tumor immunosuppression 22. In control cells, there is a main AHR enrichment peak at the promoter of BACH2. Meanwhile, there are four AHR peaks in the upstream intergenic region of the BACH2 gene. As^3+^ treatment abrogated these intergenic AHR peaks but enhanced the peak at promoter (Figure 4C). The shift or re-distribution of AHR peaks from distal intergenic and intron regions to the proximal promoter and 5'-UTR in the cells treated with As^3+^ may reflect the fact that As^3+^ may affect transcription of the genes and interaction of AHR with the transcriptional machinery, and the latter might either blunt the expression of some genes or enforce expression of other genes.

As a transcription factor, AHR can either enhance or repress gene transcription through direct binding to the consensus AHR binding motifs at promoter or distal enhancer elements that interact with promoter through long-range interactions in three-dimensional configuration. Previous studies using TCDD-treated MCF-7 cells found that AHR can bind to the AHR response element (AHRE) with a conserved sequence of 5'-GCGTG-3' or 5'-GTGCGTG-3' 23. Known Motif search using the findMotifsGenome program of the HOMER package for the 200bp surrounding the apex of AHR peaks in ChIP-seq revealed that both control and As^3+^-treated cells have a strong prevalence of the AHR binding element, 5'-CACGCAA-3' (Figure 4D). However, De Novo Motif search found that the 5'-GCGTG-3', the AHRE identified by Lo and Matthews 23 and is partially complementary to the Known Motif of AHR, is enriched for the AHR peaks in control cells, while 5'-C/TACGC-3', highly homologous to the complementary sequence of the Known Motif of AHR, is enriched among the AHR peaks in the cells treated with As^3+^. There are also some subtle shifts of element selections between control and As^3+^-treated cells in both Known Motif and De Novo Motif searches. In control cells, some AHR peaks contain elements of Fra1, JunB, ATF3, HIF1β, Nrf2, RUNX, TEAD3, MAFA, TCF21, etc, whereas the AHR peaks in the As^3+^-treated cells have elements of Fra1, Fosl2, BATF, HIF1β, Nrf2, AP-1, CEBPG, SOX8, NFIC, etc (Figure 4D). Interestingly, SOX8 has been shown to be biasedly expressed in testis around the time of male sex determination 24, which possibly corroborates with our recent findings showing long-term treatment of the cells with As^3+^ diminishes histone methylation markers of H3K4me3, H3K9me3 and H3K27me3 in chromosome Y 25. The differences in the selection of AHR binding motif between control and As^3+^ treated cells may support the observed shifts of AHR binding on the genome in response to As^3+^ and reflect the nature that As^3+^ treatment induces new selection and binding of AHR on the genome for transcriptional regulation of a subset of target genes.

**Figure 5:**
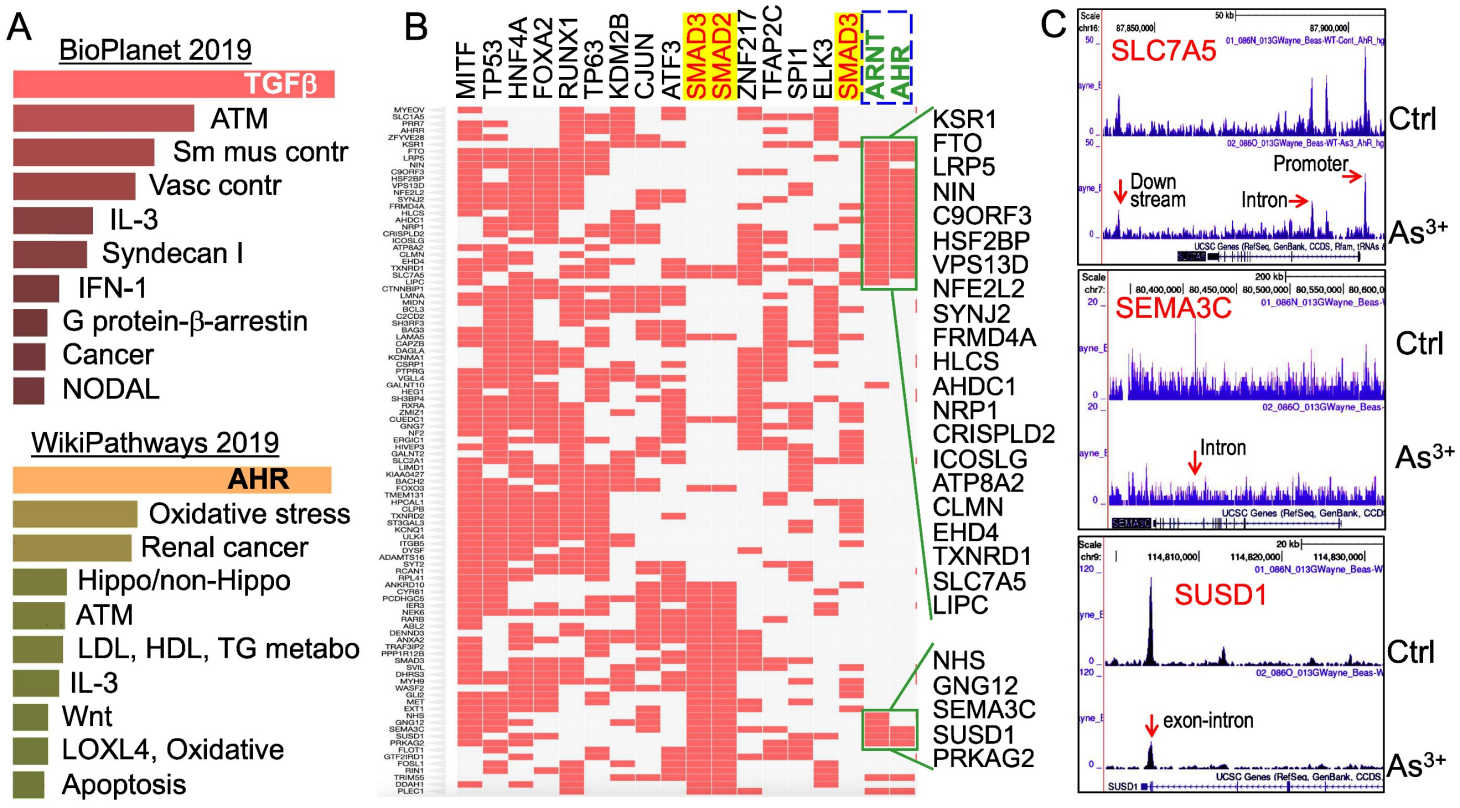
Pathway analysis of the genes with a reduced AHR binding in the As^3+^-treated cells. (A) BioPlanet 2019 (top) and WikiPathways 2019 pathway analyses for 507 genes that showed significant reduction of AHR binding in cellular response to As^3+^. (B) ChIP-seq Enrichment Analysis (ChEA) showed coordinated regulation of the indicated transcription factors for those genes with reduced AHR binding. (C) Genome browser screenshots showing the pattern of AHR enrichment peaks on the gene loci of SLC7A5, SEMA3C and SUSD1 that exhibited reduced AHR binding induced by As^3+^.

**Multiple signaling pathways are regulated by As^3+^ through AHR down-regulation**. To understand what the main targeting genes are in different signaling modules that are regulated by As^3+^ through down-regulation of AHR binding as determined by ChIP-seq, we selected a total of 507 genes that showed notable decrease of AHR binding in the cells treated with As^3+^ for Enrichr Pathway analysis. Since As^3+^ is a known toxicant and carcinogen for mammalian cells, we first analyzed those genes that showed significant enrichment of AHR in control cells but reduced AHR enrichment in the As^3+^-treated cells using BioPlanet2019, an integrated platform for exploring the cellular pathways in response to toxicological chemicals, drugs and xenobiotics. The top-ranked pathways in this analysis include TGFβ signaling, ATM-dependent DNA damage response, and smooth muscle and vascular smooth muscle contraction (Figure 5A, top panel). Considering the fact that AHR signaling is also a critical regulator for metabolism, we also applied WikiPathways analysis that focuses on the molecular pathways of metabolism, cell growth and carcinogenesis for these genes showed reduced AHR binding induced by As^3+^. As expected, AHR is the most enriched pathway in this analysis, followed by pathways of oxidative stress, renal cell carcinoma, Hippo and non-Hippo, ATM dependent DNA damage, and lipid metabolism for LDL, HDL and TG (Figure 5A, bottom panel). The effect of As^3+^ and AHR on lipid metabolism supports our recent metabolomic study showing that As^3+^ enhances lipids biosynthesis and catabolism 26.

It has been well-recognized that multiple transcription factors, rather than a single transcription factor, is required for the transcription of any given genes. To determine which transcription factors possibly concert with AHR in cellular response to As^3+^, we then performed ChIP-seq Enrichment Analysis (ChEA) that covers more than two hundred transcription factors. As depicted in Figure 5B, in addition to AHR and ARNT, an AHR interacting protein in the AHR heterodimer, transcription factors of MITF, TP53, FOXA2, ATF3, SMAD2, SMAD3, cJun, etc, are over-represented among those genes that showed reduced AHR binding in response to As^3+^. The over-representation of SMAD2 and SMAD3, the central transcription factors mediating the canonical TGFβ signaling pathway 27, supports the observed top-ranking of TGFβ signaling in the BioPlanet2019 assay (Figure 5A) and the observed interplay or mutual antagonism between AHR and TGFβ signaling 28, 29. Some differences in AHR binding patterns were noted for the genes that are co-regulated by TP53, SMAD2 and SMAD3 (Figure 5B). The AHR enrichment peaks in ChIP-seq can be found in gene promoter, intron, exon, upstream, or downstream, as exampled for SLC7A5, SEMA3C and SUSD1 (Figure 5C).

**Figure 6:**
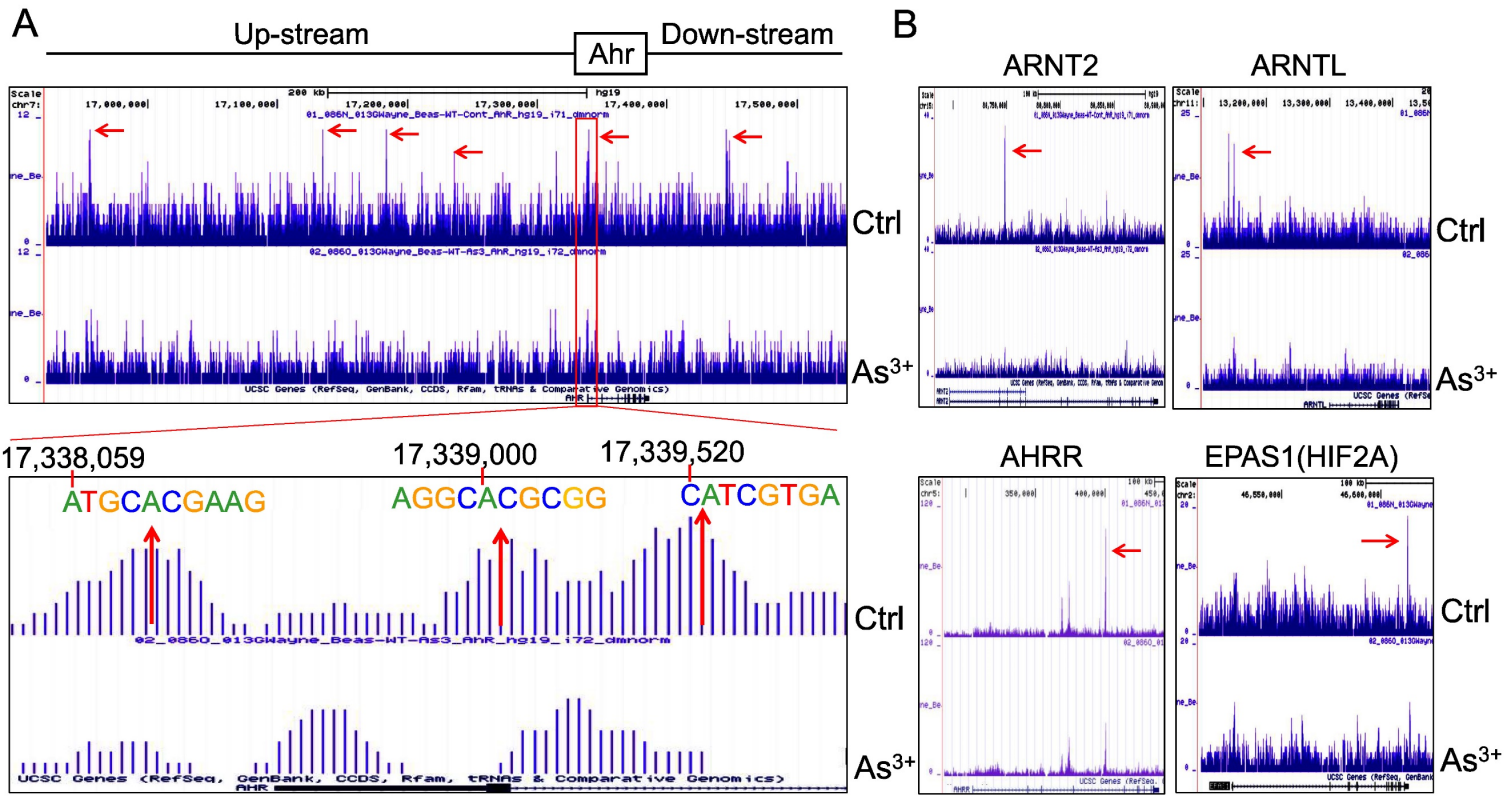
Forward feedback of AHR and the key AHR pathway genes. (A) AHR enrichment peaks were found on the AHR gene locus. Bottom panel shows consensus AHR binding motifs within the AHR peak in the promoter region of the AHR gene. (B) As^3+^ treatment reduced AHR binding on the genes of ARNT2, ARNTL, AHRR, and EPAS1.

**AHR pathway is featured with a self-feedback regulation**. It had been reported that AHR may sustain itself through the IDO1-kynurenine-AHR-IDO1 feedback loop in leukemia cells and human embryonic stem cells 30, 31. Meanwhile, it has been recently shown that AHR appears to be able to enhance the chromatin accessibility on its own gene locus in gut group 2 innate lymphoid cells (ILC2s) 17. In our ChIP-seq analysis, we noted multiple AHR enrichment peaks in AHR gene locus. Although these AHR peaks are relatively weak comparing those with other genes, AHR binding elements were identified for some of these peaks, esp. the peaks at the promoter region of AHR (Figure 6A). This indicates that AHR may be self-regulated through binding to its own gene promoter or enhancers. The AHR enrichment peaks were also found on the genes encoding AHR-interacting proteins, including ARNT2, ARNTL, AHRR, and EPAS1 (HIF2A) (Figure 6B). Treatment of the cells with As^3+^ also reduced the overall enrichment level of AHR on these AHR family genes.

**Figure 7:**
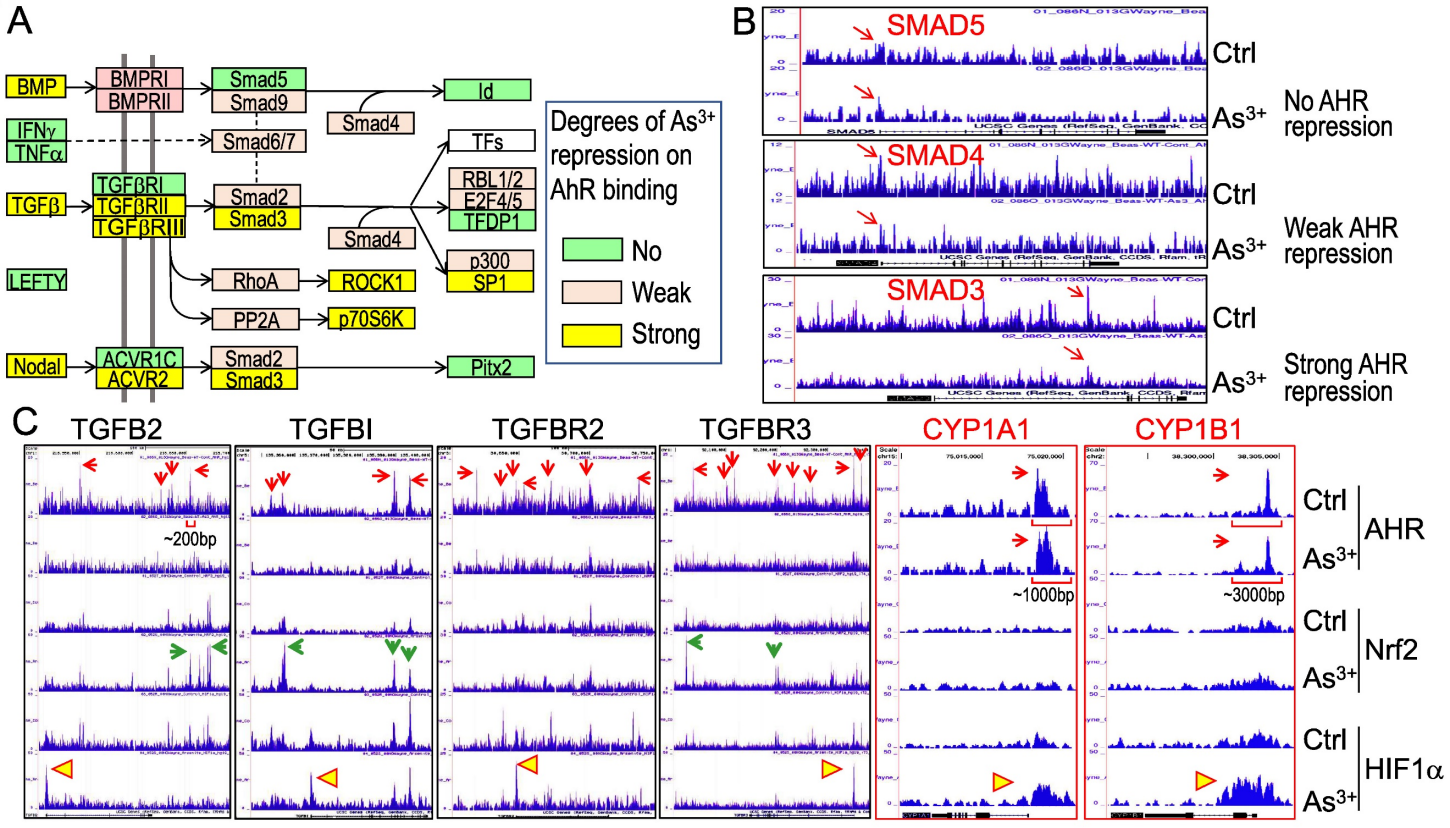
As^3+^ diminishes AHR binding to the TGFβ signaling pathway genes. (A) Diagram of the simplified TGFβ signaling and summary of the AHR binding status on these genes. (B) Examples show no repression, weak repression and strong repression of AHR binding to the different TGFβ signaling genes induced by As^3+^. (C) Genome browser screenshots showing As^3+^ reduces AHR binding but enhances Nrf2 and/or HIF1α binding to these indicated TGFβ pathway genes. For comparison, the enrichment patterns of AHR, Nrf2 and HIF1α on two classical AHR targeting genes, CYP1A1 and CYP1B1 were shown also. Horizonal square brackets indicate the width of AHR peak bases of the genes of TGFB2, CYP1A1 and CYP1B1. Red arrows: AHR ChIP-seq peaks; green arrows: As^3+^-enriched Nrf2 ChIP-seq peaks; yellow triangles: As^3+^-enriched HIF1α ChIP-seq peaks.

**As^3+^ treatment weakens AHR binding to the genes in TGFβ signaling pathway**. Pathway analysis unraveled that TGFβ signaling is one of the most important regulators for these genes with diminished binding of AHR in response to As^3+^ (Figures 5A & 5B). To further understand how AHR affects the TGFβ signaling, we retrieved the key genes in TGFβ signaling and evaluated the degree of AHR repression in the cells treated with As^3+^ by scaling the highest peak of AHR in Genome Browser of ChIP-seq data for each of the tested genes. Several genes in this pathway, including BMP, TGFβ, TGFβRII/III, Smad3, etc., exhibited strong repression (>50% reduction of the peak height) of AHR induced by As^3+^. Other genes, such as BMPRI, Smad2, Smad4, and others, showed weak repression of AHR induced by As^3+^ (>5% but <50% of reduction of the peak height) (Figures 7A & 7B). By incorporating the ChIP-seq results of Nrf2 and HIF1α from the same cells, we observed a generally opposite effect of As^3+^ on the chromatin binding of AHR and Nrf2 or HIF1α. For most of these TGFβ signaling genes, treatment of the cells with As^3+^ abrogated AHR binding, but enhanced Nrf2 and/or HIF1α binding on these genes (Figure 7C). These results suggested that it is very likely that As^3+^-induced Nrf2 and/or HIF1α promotes the expression of these TGFβ pathway genes, whereas AHR is inhibitory for the transcription of these genes. It is also indicative that despite a concerted regulation of AHR and Nrf2 in the metabolism and detoxification of xenobiotics, these two transcription factors may be mutually antagonistic for the expression of genes in TGFβ signaling.

We also examined the AHR binding status on the genes of the classic AHR targeting genes, including CYP1A1, CYP1B1 and others. Although As^3+^ treatment also reduced AHR binding to the genes of CPY20A1, CYP26B1, CYP27A1, CYP27C1, and the CYP2 cluster (data not shown), no significant reduction of AHR binding induced by As^3+^ was noted on the genes of CYP1A1 and CYP1B1 in ChIP-seq analysis (CYP1A1 and CYP1B1 panels in Figure 7C). The base width for the majority of the AHR peaks on the genes of TGFβ and other oncogenic signaling is around 200bp. The base width of the AHR peaks in the promoters of CYP1A1 and CYP1B1, in contrast, is between 1000 to 3000bp, which reflects the nature of multiple AHR binding elements (AHREs) in these peak regions. Indeed, it had been shown that both mouse and human CYP1A1 and CYP1B1 genes contain 8 to 11 AHREs in the regions corresponding to the AHR peaks in ChIP-seq in this report 32-34. Such an unusual enrichment of AHREs may render the resistance to As^3+^-induced reduction of AHR binding to CYP1A1 and CYP1B1 genes.

**Figure 8:**
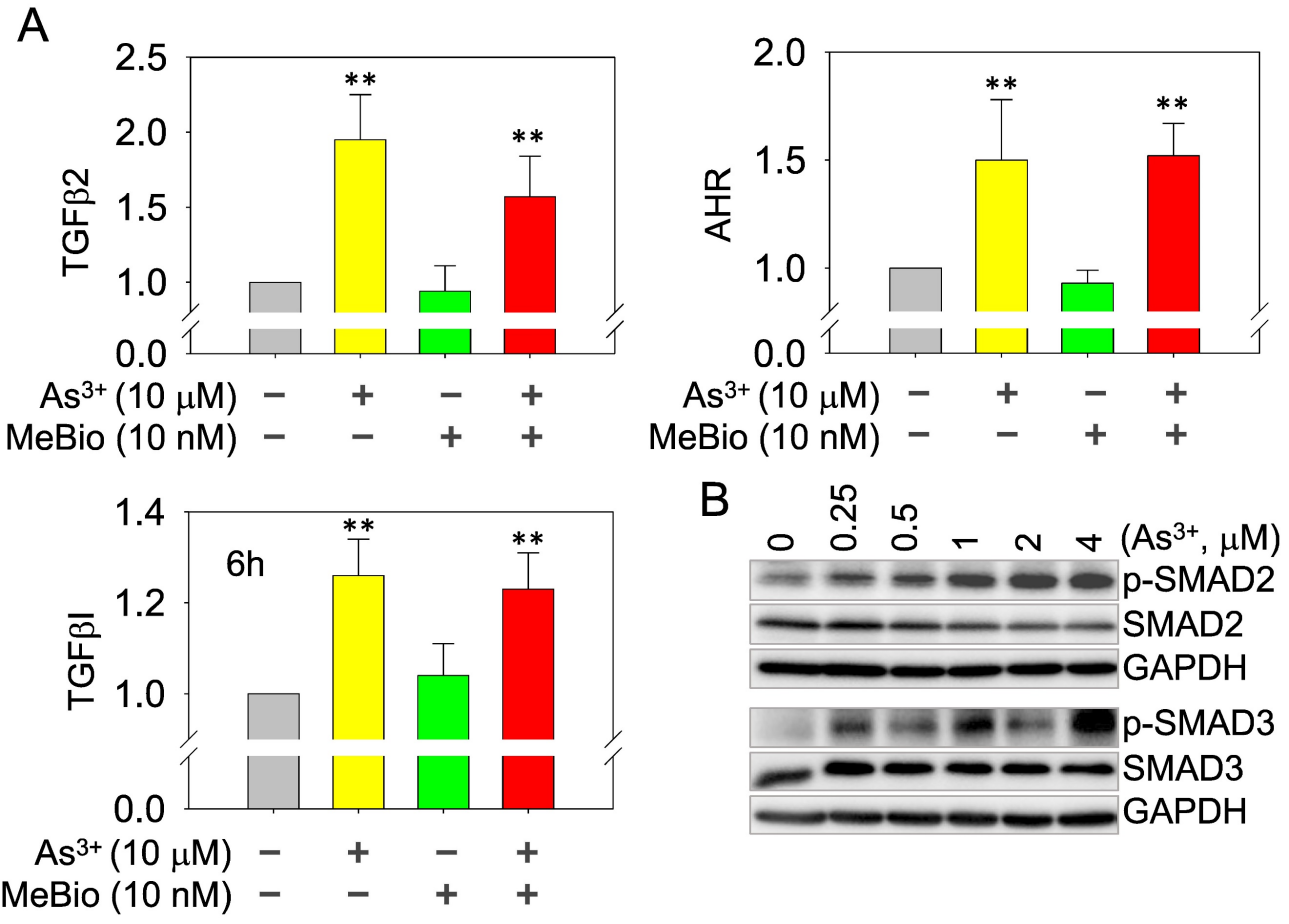
As^3+^ induces TGFβ signaling and AHR genes. (A) Quantitative real-time PCR for the mRNAs of TGFβ2, TGFβI, and AHR using total RNAs extracted from the control cells, cells treated with 10 μM As^3+^, 10 nM MeBio, or both for 6h. **: p **≤** 0.001 as compared with the control cells without any treatment. (B) Dose-dependent phosphorylation of SMAD2 and SMAD3 induced by As^3+^.

**As^3+^, but not the activated AHR, enforces TGFβ signaling**. Whether AHR is a promoter or repressor for TGFβ signaling had been debated in the past years 35, 36. Depending on the tissues or cell types and the use of different AHR agonists or antagonists, AHR can either induce or inhibit the gene expression of the TGFβ signaling members. In real-time PCR, we found As^3+^ is very potent in inducing the mRNA expression of TGFβ2, TGFβI, and AHR. The AHR agonist MeBio, on the other hand, neither induces nor inhibits the expression of these mRNAs (Figure 8A). A similar observation is that MeBio failed to affect As^3+^-induced expression of these tested genes in co-treatment experiment. The potency of As^3+^ on TGFβ signaling is additionally confirmed by the dose-dependent enhancement of Smad2 and Smad3 phosphorylation (Figure 8B).

**Figure 9:**
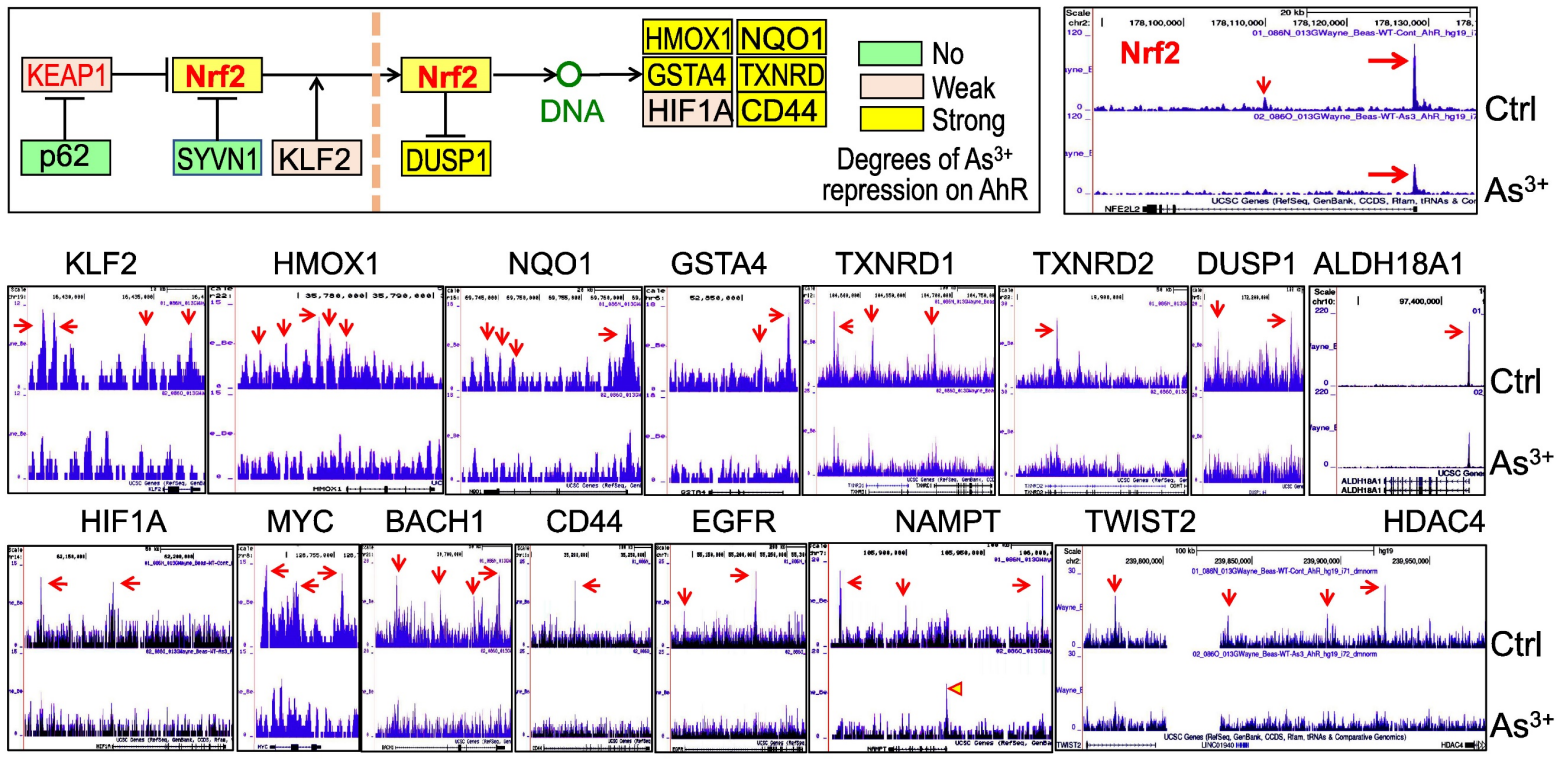
Diminished AHR binding to the key Nrf2 pathway genes and some Nrf2-dependent oncogenes in the cells treated with 1 μM As^3+^ for 6h, followed by ChIP-seq analysis. Up-left penal shows diagram of Nrf2 signaling and summary of AHR binding status on these genes in response to As^3+^. Middle row: AHR binding patterns on these Nrf2 pathway genes in control cells and As^3+^-treated cells. Bottom row: AHR binding patterns on these Nrf2-dependent oncogenes in control cells and As^3+^-treated cells.

**Diminished AHR binding to the Nrf2 signaling genes in cellular response to As^3+^**. We had recently shown that Nrf2 signaling played critical role in As^3+^-induced glycolytic metabolism and the generation of the cancer stem-like cells 10. Others had also demonstrated the oncogenic role of Nrf2 in many types of human cancer 37. Some earlier reports suggested an AHR dependent expression of Nrf2 mRNA and protein 38, 39. To determine what role of AHR played on the As^3+^-induced Nrf2 activation, we assessed AHR enrichment on these Nrf2 signaling pathway genes using the ChIP-seq data from the control cells and the As^3+^-treated cells. Resembling to the TGFβ pathway genes, As^3+^ treatment decreased AHR binding on most of the Nrf2 pathway genes (Figure 9). In control cells, there is a major AHR enrichment peak in the Nrf2 gene promoter in ChIP-seq, and a minor AHR peak in the first intron. Both peaks were substantially eroded in the As^3+^-treated cells (Figure 9, top right). The same is true for most of the Nrf2 pathway genes (Figure 9, middle row) and those Nrf2-dependent oncogenic genes, including HIF1α, MYC, BACH1, CD44, EGFR, NAMPT, TWIST2, and HDAC4 (Figure 9, bottom row). Among these Nrf2-dependent oncogenic genes, the distribution of AHR peaks on the gene locus of NAMPT is unique. As^3+^ reduced the peaks in upstream, gene body and downstream, but amplified the peak at promoter. These results, thus, suggest that it is unlikely that AHR can promote or intensify the expression of Nrf2, Nrf2 pathway and Nrf2 target genes under the condition of the cells exposed to As^3+^.

**Figure 10:**
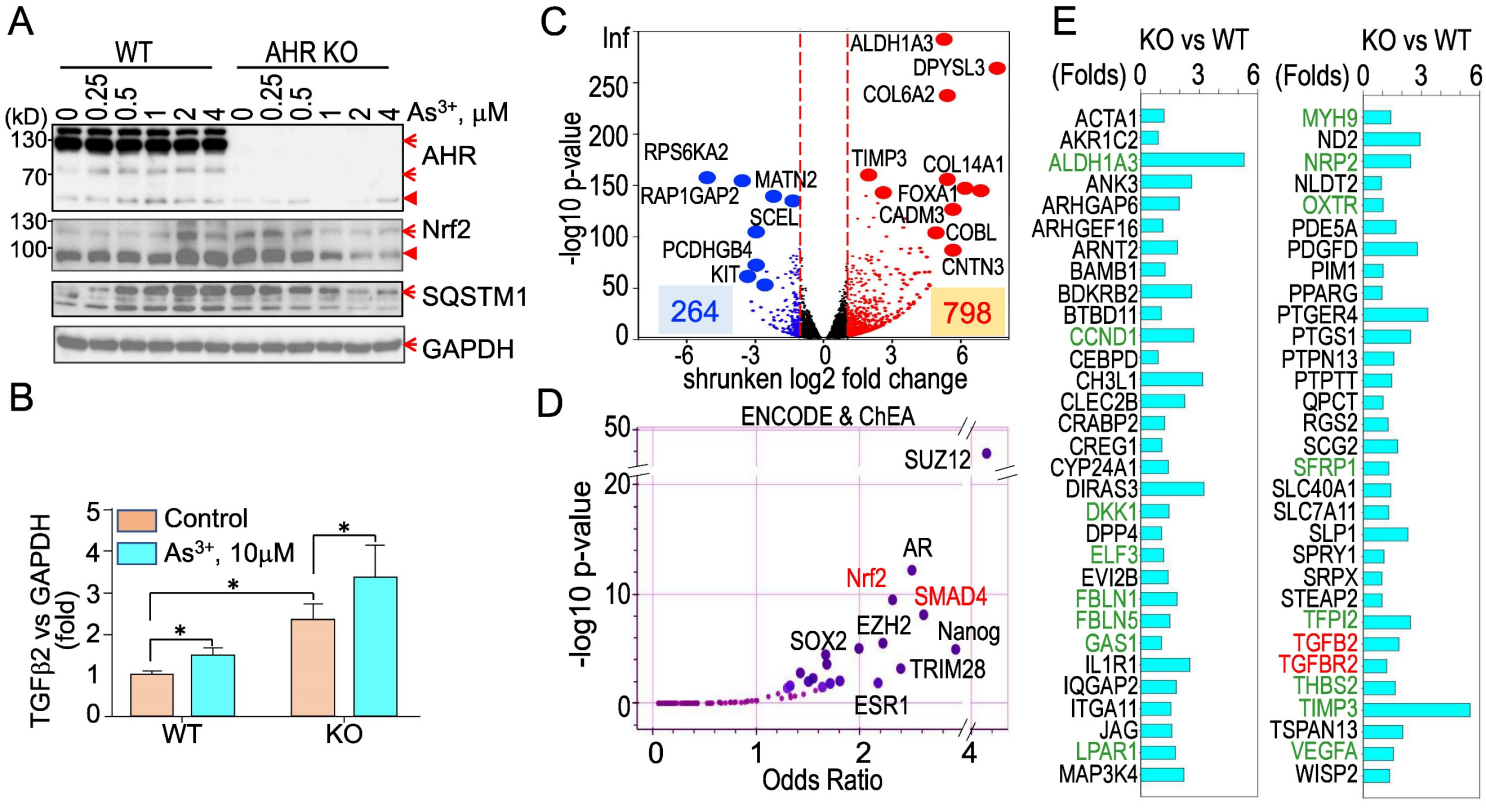
Knockout of AHR through CRISPR-Cas9 gene editing enforces TGFβ signaling. (A) Wild type (WT) and AHR knockout (KO) cells were treated with the indicated concentrations of As^3+^ for 6h followed by Western blotting to determine the protein levels of AHR, Nrf2, SQSTM1, and GAPDH. Red arrows denote the specific bands of the proteins; filled red triangles indicate non-specific bands. (B) Quantitative real-time PCR for detecting TGFβ2 mRNA from the WT and AHR KO cells under control or treated with 10 μM As^3+^ for 6h. Data are expressed as mean ± SD. *: p < 0.05 in control vs As^3+^. (C) Volcano plot of RNA-seq showing differential expression of genes in AHR KO cells vs WT cells. In the KO cells, 264 genes are downregulated and 798 genes are upregulated. (D) ENCODE & ChEA assay of the 798 upregulated genes in the KO cells. (E) List of TGFβ-targeting genes that are upregulated in the AHR KO cells. Genes in greens are known oncogenes for carcinogenesis and formation of the cancer stem-like cells.

**Knockout of AHR enhances basal Nrf2 activation and the As^3+^-induced TGFβ signaling**. To strengthen the evidence showing the regulatory role of AHR on TGFβ and other biological or biochemical responses of the cells, we took CRISPR-Cas9 gene editing approach to knockout AHR in BEAS-2B cells. After screening for the AHR knockout clones of the cells subjected to two different sgRNAs targeting exon1 and exon2, respectively, in CRISPR-Cas9 editing, Western blotting was performed to measure the protein levels of AHR, Nrf2, SQSTM1, and GAPDH in a randomly selected wild type (WT) cell clone and an AHR knockout (KO) cell clone treated with 0 to 4 μM As^3+^ for 6h. The success rates of AHR knockout by sgRNA1 and sgRNA2 are 10% and 40%, respectively (date not shown). As expected, no AHR protein was detected in the AHR KO cells. A notable dose dependent induction of Nrf2 and SQSTM1 by As^3+^ was observed in the WT cells. No induction of Nrf2 and SQSTM1 by As^3+^ was detected in the KO cells. However, a basal level elevation of Nrf2 and SQSTM1 was noted in the KO cells, indicating AHR is indeed inhibitory for Nrf2, at least at the basal condition (Figure 10A). To determine whether knockout of AHR affects the expression of TGFβ family members, total RNAs were prepared from both the WT and KO cells for real-time PCR of cytokine TGFβ2. The AHR KO cells showed a pronounced enhancement of both basal and As^3+^-induced TGFβ2 expression (Figure 10B), which clearly suggests that AHR is inhibitory on the TGFβ signaling pathway. To additionally address the negative regulation of AHR on TGFβ and other signaling, transcriptomics through RNA-seq was conducted using mRNAs extracted from three WT and three AHR KO cell clones. A total of 264 genes decreased and 798 genes increased in the AHR KO cells relative to the WT cells from RNA-seq (Figure 10C). ChIP-seq Enrichment Analysis (ChEA) for these up-regulated genes in the KO cells demonstrated over-representation of transcription factors of SUZ12, AR, Nrf2, SMAD4, as well as SOX2 and Nanog important for the stem cells and cancer stem cells (Figure 10D). SMAD4 can form heterodimers with phosphorylated SMAD2 or SMAD3 for the transcription of the TGFβ-targeting genes 40. Indeed, among these upregulated genes in the AHR KO cells, in addition to TGFβ2 and TGFBR2, around 60 TGFβ targeting genes are identified (Figure 10E). Many of these TGFβ targeting genes are known genes involved in carcinogenesis or the generation of the cancer stem-like cells (green colored in Figure 10E).

**Figure 11:**
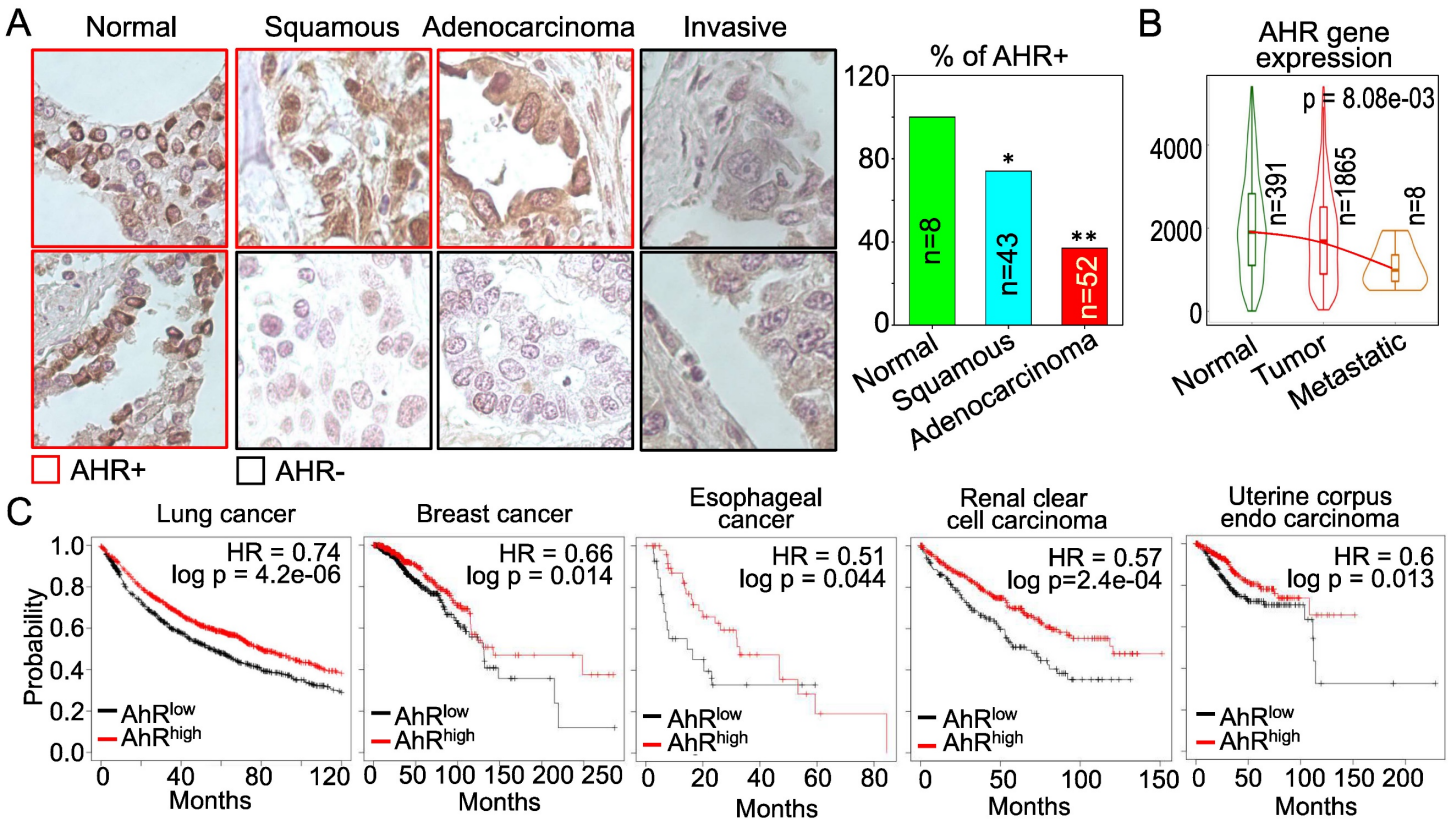
AHR is tumor suppressive in human lung cancer and other cancers. (A) Immunohistochemistry (IHC) of AHR protein expression in normal human lung tissues and lung cancer tissues. Right panel summarized the percentage of AHR positivity in normal lung tissue and lung cancer tissues. *: p < 0.05 in Fisher exact probability test as compared to normal tissue; **: p < 0.001 in Fisher exact probability test as compared to the normal tissue. (B) Expression levels of AHR in normal lung, lung cancer and metastatic lung cancer. Data are retrieved from TNMplot database. Total case numbers (n) in each tissue category are indicated inside of the panel. (C) Overall survival probability and AHR expression status of the patients with lung cancer, breast cancer, esophageal cancer, renal clear cell carcinoma, and uterine corpus endo carcinoma. Data are retrieved from Kaplan-Meier Plotter database. Case numbers of high AHR expression (red n) and low AHR expression (black n) were marked in each panel. The high and low AHR expression of tumor samples were determined by computing upper and lower quartiles of AHR expression among these samples and selecting the best cutoff option in the Kaplan-Meier Plotter program.

**AHR possesses tumor suppressor-like property in human cancers**. The above data showing inhibitory role of AHR on TGFβ and Nrf2 are indicative that AHR may be tumor suppressive, rather than oncogenic. However, these cell-based data need to be validated by clinical evidence of human cancers. For that purpose, we examined AHR expression in human lung cancer tissue samples along with normal lung tissues by immunohistochemistry (IHC). Strong positivity (100%) of AHR protein expression was observed in all normal lung tissues. However, the AHR positive rate is much lowered among the tissue samples of lung squamous cell carcinoma, lung adenocarcinoma and invasive lung cancer (Figure 11A). This observation is corroborated by the degree of AHR gene expression in normal lung, lung tumors and metastatic lung tumors in TNMplot database that contains 391 cases of normal lung tissues, 1865 cases of lung cancer tissues and 8 cases of metastatic lung tumor tissues (Figure 11B). AHR expression is declined in lung tumors relative to the normal lung tissue, which is further diminished in the metastatic lung tumors. The cancer patient survival data provided another layer of vigorous support for the tumor suppressor-like activity of AHR. As shown in Figure 11C, high level of AHR in cancers of lung, breast, esophagus, kidney, and uterus predicts better overall survival of the patients, whereas survival probability of lower AHR cancers is significantly poorer. Thus, it is unequivocal that AHR is tumor suppressive, rather than oncogenic, at least in human lung cancer. These data also suggest that one of the carcinogenic mechanisms of As^3+^ may achieved through antagonizing the tumor suppressor-like activity of AHR.

## Discussion

Accumulating data provided unequivocal evidence linking environmental As^3+^ to human cancers. As^3+^ is highly capable of inducing oxidative stress, activation of protein kinases and oncogenic transcription factors, perturbation of immune responses, reprogram of the metabolism and epigenetics, and/or damages of DNA in the genome 9. The current report unraveled a new mechanism of As^3+^ carcinogenesis, which suggests that As^3+^ is inhibitory for the tumor suppressor-like character of AHR, followed by the amplification of the oncogenic pathways of TGFβ, Nrf2 and others. The tumor suppressor-like feature of AHR was additionally substantiated by the diminished expression of AHR in human lung cancer tissues and poorer prognosis of the patients with lower AHR tumors.

The first identified function of the ligand activated AHR is the transcriptional activation of several xenobiotic phase I and phase II metabolizing enzymes. Some of these enzymes are able to convert inert chemical carcinogens to the nucleophilic molecules that may form adducts with DNA or directly damage the genomic DNA. Meanwhile, the most widely studied and the most potent AHR ligands, including TCDD and B[a]P, are well-documented human carcinogens 41. It was shown that the carcinogenicity or tumorigenicity of TCDD is in an AHR dependent manner 42, 43. Studies by Anderson et al suggested that constitutively active AHR facilitates the development of stomach tumors in mice 44. Others showed that the expression level of AHR in invasive tumors is higher than the non-invasive tumors 45, 46. AHR appears to be essential for the skin carcinogenesis induced by UV radiation and chemical carcinogens 47. In addition, knockout of AHR in fibroblasts diminishes the tumorigenicity of the cells along with a down-regulation of proto-oncogene VAV3 in a xenograft model 48. It is speculated that the pro-carcinogenic or tumorigenicity of AHR might be achieved through its regulation on cell-cell contact, cell proliferation, dedifferentiation, and motility 41. Accordingly, AHR was considered as an oncogenic transcription factor during carcinogenesis.

Despite implications of AHR as an oncogenic factor, the tumor suppressor-like property of AHR had also been uncovered in several reports using a variety of experimental models. TCDD is the most widely used AHR agonist and an earlier study suggested an inhibition of TCDD on the metastasis of mouse breast cancer cells *in vivo* 49. The key evidence of tumor suppressor-like activity of AHR was provided by AHR gene knockout model of mouse that showed increased liver tumorigenesis induced by diethylnitrosamine (DEN) in the AHR knockout mice 50. This notion was supported by the increased incidence rate of premalignant colon cancer lesion induced by high-fat diet in mouse with intestinal-specific AHR knockout 51. Of note, AHR was found to be protective in suppression of the mutagen azoxymethane (AOM)-induced colitis-associated colorectal carcinoma (CAC) 52. Deletion of AHR specifically in gut epithelial cells enforces proliferation of the gut epithelial stem cells but compromises differentiation of these stem cells into goblet and enterocytes, leading to the development of AOM-induced CAC. Similarly, an earlier study suggested that AHR is capable of destabilizing β-catenin, a well-documented proto-oncogene, through its ligand-dependent E3 ubiquitin ligase activity 53. Even with the known carcinogenicity of some AHR agonists from the environmental sources, there is evidence suggesting AHR independent induction of oncogenes, including c-fos and c-jun, in cultured cells 54. In neuroblastoma, high level of AHR expression not only correlated with the less malignancy of the tumors but associated with better survival outcomes of the patients, indicating the tumor suppressor-like activity of AHR 55. This notion was further supported by the facts that enforced expression of AHR suppresses neuroblastoma progression *in vivo* and kynurenine, an endogenous agonist of AHR derived from tryptophan metabolism 56, inhibits proliferation and promotes differentiation of the neuroblastoma cells 55. Most recently, Phillips et al 57 generated AHR and p53 double knockout mice and found higher incidence rate of thymic lymphoma, leukemia, sarcomas, and gastrointestinal tract inflammation in the AHR^-/-^p53^-/-^ mice relative to the AHR^+/+^p53^-/-^ mice. All these findings, thus, unequivocally suggest that AHR may be tumor suppressive, rather than oncogenic in many types of cancers.

If AHR is tumor suppressive, critical questions to be answered are what mechanism mediates this tumor suppressor-like capacity of AHR, is this AHR ligand dependent or independent, and whether this effect is limited to specific tumor types and stages. Studies in Sonic Hedgehog (SHH) medolloblastomas unraveled that depletion of AHR drives tumor growth due to elevated activation of the TGFβ signaling and the enrichment of SOX2 positive cancer stem-like cells. Pharmacological intervention of the TGFβ signaling was sufficient to inhibit the proliferation and promote the differentiation of the Ahr^-/-^ cancer-propagating cells of the SHH medulloblastomas 28. In human glioma, it had been shown that high activation of TGFβ signaling confers poor prognosis of the patients and promotes tumor cells proliferation 58, which is very likely due to the enhanced JAK-STAT pathway and self-renewal of the cancer stem-like cells 59.

The data in this report clearly indicate that AHR is a negative regulator for the TGFβ signaling and As^3+^ disrupts the transcriptional activity of AHR, leading to the consequent enhancement of TGFβ signaling as well as other oncogenic signaling. Transcriptional repression of AHR on TGFβ or TGFβ pathway genes was also demonstrated in smooth muscle cells derived from AHR gene knockout mice 60. In WT cells, AHR represses expression of TGFβ and the genes involved in modulating and processing of the TGFβ signaling. Knockout of AHR elevated the mRNA levels of TGFβ, TGFβ-related genes, and CYP1B1 induced by TCDD. The inhibitory effect of AHR on TGFβ and the associated extracellular matrix (ECM)-related genes was further observed in the fibroblast cells from the AHR knockout mice 61. The tumor suppressor-like property of AHR by repressing TGFβ signaling was most recently elucidated in the anti-metastasis of AHR in human lung cancer cells 62. By an unbiased shRNA screen in H1975 human lung adenocarcinoma cells, AHR was uncovered as a major anti-metastatic factor through restraining the expression of TGFβ and some genes in EMT and invasion of the tumor cells. In addition to the inhibitory effect of AHR on TGFβ signaling, AHR may also act as a repressor for the proliferation of mesenchymal stem cells (MSCs) in bone marrow 63. Activation of AHR by some agonists prevented the proliferation and self-renewal of MSCs, whereas AHR antagonists or siRNA-mediated silence of AHR stimulate proliferation of MSCs. There is evidence indicating that repression of AHR on MSCs was associated with a reduced inflammation upon tissue injury. Mice with AHR knockout exhibited an impaired migratory potential of MSCs and a heightened lung inflammation in response to allergen exposure 64. Furthermore, AHR may directly participate in the anti-inflammatory regulation through antagonizing NF-κB, a master transcription factor for a wide array of inflammatory cytokines 65.

Our data suggested a marginal induction of AHR protein but a substantial repression of the transcriptional or chromatin binding activity of the AHR in cellular response to As^3+^. Whereas the induction of AHR might be partially dependent on the activation of Nrf2 signaling, it remains to be fully elucidated at the present on how As^3+^ impairs the transcriptional activity of AHR. Following exogenous or endogenous ligand binding and translocation from cytoplasm to nucleus, AHR will dissociate from chaperone proteins and in turn form heterodimers with aryl hydrocarbon receptor nuclear translocator (ARNT) for active transcription of the AHR targeting genes. There are several possibilities that may explain how As^3+^ interferes with the AHR activity in the nucleus. First, As^3+^ may directly disrupt the formation of AHR-ARNT heterodimers through altering the 3D structures of the AHR or ARNT protein. Second, since ARNT is also an active partner of HIF1α complexes and As^3+^ is highly capable of activating the HIF1α signaling, it is very likely that activated HIF1α will compete with AHR for ARNT interacting, leading to inhibition of the AHR transcriptional activity. Third, some AHR negative regulators, such as AHRR, may be induced by As^3+^, to interrupt the DNA binding and transcriptional activity of AHR. Lastly, a number of other transcription factors share AHREs and other AHR binding elements (Figure. 4D) for DNA binding, and these transcription factors, such as Fra1, AP1, ATF3, Nrf2, etc., may possess higher affinity toward AHREs and other AHR binding elements. Induction of these transcription factors by As^3+^, accordingly, will result in replacement of AHR at these binding sites, leading to an overall reduction of DNA binding and transcriptional activity of AHR.

A daunting question to be asked is why AHR is a transcriptional activator for the classic AHR target genes, such as p450 CYP1A1 and CYP1B1, but acts as a transcriptional repressor for the genes in TGFβ and other oncogenic pathways. One of the possible answers to this question is the number, location and surrounding sequences of the consensus AHR binding elements (AHRE), also called xenobiotic response elements (XRE) or dioxin response elements (DRE). It has been known that both CYP1A1 and CYP1B1 genes have multiple, 8 to 11, AHREs in the promoter or upstream of the genes 32-34. It is possible, thus, that these multiple AHREs may enforce the transcriptional activation of the AHR. Indeed, no reduction or only marginal reduction of AHR binding on the CYP1A1 and CYP1B1 genes was noted in the As^3+^-treated cells (Figure 7C). In contrast, The AHR binding peaks in the genes of TGFβ signaling pathway and other oncogenic pathways only contain one or two AHREs in either gene body or down-stream of the genes. It is very likely that AHR binding to these genes may interact with other transcription factors or transcriptional repressors to confer transcription repression. The different or opposite transcriptional regulation of AHR on CYP1A1 and oncogenic genes was also observed in human glioblastoma cell lines 66. Either siRNA silencing or CRISPR-Cas9-based knockout of AHR prevented expression of CYP1A1, but amplified the expression of MMP9, CXCL12 and CXCR4 that contribute to the tumorigenesis and metastasis of the cancer cells.

Despite both pro- and anti-tumorigenic activities of AHR had been uncovered in a wide spectrum of experimentations or clinical observations as discussed above, it is difficulty at the present to characterize when and how AHR is oncogenic or tumor suppressive. Diverse factors, including types and abundance of AHR agonists and antagonists, the degrees of AHR activity, synchronous and asynchronous signaling pathways, physiological and pathological status of the cells or tissues, etc., may impact the character switch of AHR in carcinogenesis. One of the possible scenarios is that provocation of AHR by certain environmental pollutants is oncogenic through enforcing malignant transformation of the normal cells due to the metabolic activation of the inert chemicals by the downstream enzymes of AHR, mostly the p450 family members. In the transformed or cancer cells, in contract, AHR is tumor suppressive through its negative regulation on signaling pathways of TGFβ, Nrf2, and other oncogenes. The loss of AHR expression in human lung cancer tissues as demonstrated in this report (Figure 11) supports such a hypothesis. It also raises the possibility that boosting AHR in certain types of cancers, such as lung cancer, may improve the efficacy of cancer therapies.

## Conclusion

**Figure 12:**
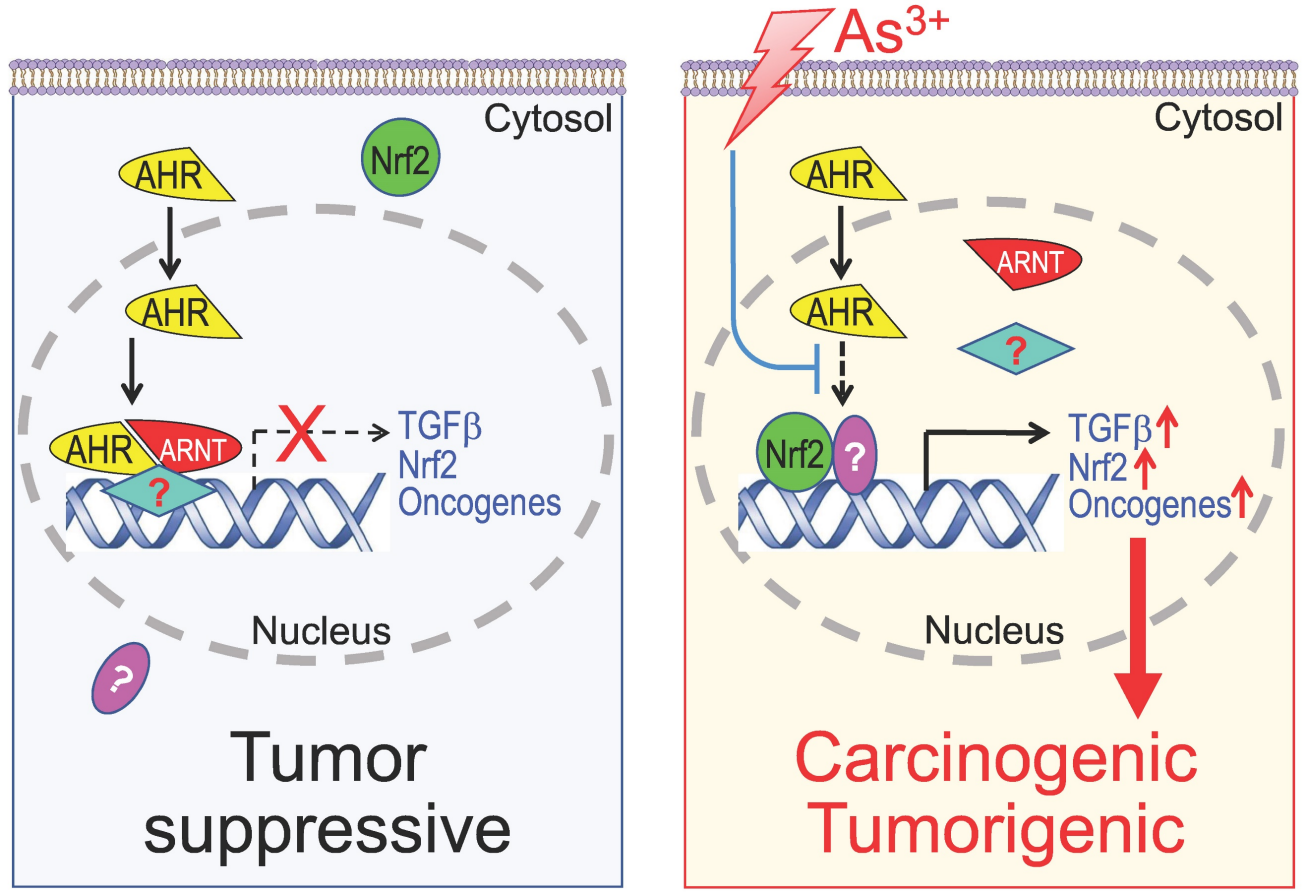
Diagram shows new mechanism of As^3+^ carcinogenesis. Under normal condition, either basal or ligand-activated AHR can translocate to nuclei where it form repressive complexes with other nuclear proteins to limit the expression of the genes in the pathways of TGFβ, Nrf2 and oncogenesis. The cells are in a state of tumor suppressive. Exposure of the cells to As^3+^ will disrupt the tumor suppressor-like activity of AHR, either through preventing the formation of repressive complexes and/or blocking recruitment of the repressive complexes to these oncogenic genes. Meanwhile, the reduced AHR binding to the DNA may also enhance the nuclear translocation and transcriptional regulation of these oncogenic transcription factors induced by As^3+^, such as Nrf2 and HIF1α, leading to active transcription and expression of oncogenes and genes in the TGFβ and Nrf2 signaling pathways, which promotes carcinogenesis or tumorigenesis.

The data from the current report provide a new support to the tumor suppressor-like property of AHR that negatively regulates the TGFβ and other oncogenic signaling in cellular response to As^3+^. There are scattering studies indicating activation of AHR signaling by As^3+^ or other arsenic compounds 67. Our data, however, clearly suggest that As^3+^ is inhibitory for the transcriptional activity of AHR through impeding the binding of AHR on the genome, although As^3+^ can slightly induce AHR protein and its nuclear translocation. ChIP-seq analysis indicated that TGFβ signaling is the most prominent pathway amplified by As^3+^ through its inhibition on AHR. Thus, interruption of the tumor suppressive-like activity of AHR, followed by the intensified TGFβ signaling, Nrf2 signaling and others, may be one of the key mechanisms of As^3+^-induced carcinogenesis (Figure 12). Such a finding may grant rational designs of new molecular targeting therapy for human cancers associated with environmental exposure to As^3+^ and other carcinogens, since AHR is highly targetable by either agonists or antagonists. There is a wide spectrum of non-carcinogenic AHR agonists, such as those naturally originated flavonoids, indole derivatives, tryptophan metabolites, etc., that may be applied for boosting the tumor suppressor-like activity of AHR.

## Abbreviations

AHR: Aryl hydrocarbon receptor
ChIP-seq: Chromatin immunoprecipitation and sequencing
ChEA: ChIP-seq enrichment analysis
CRISPR-Cas9: Clustered regularly interspaced short palindromic repeats-CRISPR associated protein9
DEN: Diethylnitrosamine
GEO: Gene expression omnibus
HIF1α: Hypoxia inducible factor 1 subunit alpha
IHC: Immunohistochemistry
KO: Knockout
MSCs: Mesenchymal stem cells
Nrf2: Nuclear factor erythroid 2-related factor 2
RPKM: Reads per kilobase of transcript per million
sgRNA: Single guide RNA
SHH: Sonic hedgehog
SHS: Strong heart study
TCDD: 2,3,7,8-Tetrachlorodibenzo-p-dioxin
TGFβ: Transforming growth factor beta
Th17: T helper cell 17
Treg: T regulatory cells
U.S. EPA: United States Environmental Protection Agency
WHO: World Health Organization
WT: Wild type
